# Defining the physiological determinants of low nitrogen requirement in wheat

**DOI:** 10.1042/BST20200282

**Published:** 2021-03-26

**Authors:** Nick S. Fradgley, Alison R. Bentley, Stéphanie M. Swarbreck

**Affiliations:** 1 The John Bingham Lab, NIAB, 93 Lawrence Weaver Road, Cambridge CB3 0LE, U.K.; 2 Department of Plant Sciences, University of Cambridge, Downing Street, Cambridge CB2 3EA, U.K.

**Keywords:** nitrogen, wheat, yield

## Abstract

Nitrogen (N) is a major nutrient limiting productivity in many ecosystems. The large N demands associated with food crop production are met mainly through the provision of synthetic N fertiliser, leading to economic and ecological costs. Optimising the balance between N supply and demand is key to reducing N losses to the environment. Wheat (*Triticum aestivum* L.) production provides food for millions of people worldwide and is highly dependent on sufficient N supply. The size of the N sink, i.e. wheat grain (number, size, and protein content) is the main driver of high N requirement. Optimal functioning of temporary sinks, in particular the canopy, can also affect N requirement. N use efficiency (i.e. yield produced per unit of N available) tends to be lower under high N conditions, suggesting that wheat plants are more efficient under low N conditions and that there is an optimal functioning yet unattained under high N conditions. Understanding the determinants of low N requirement in wheat would provide the basis for the selection of genetic material suitable for sustainable cereal production. In this review, we dissect the drivers of N requirement at the plant level along with the temporal dynamics of supply and demand.

## Introduction

**Figure 1.**
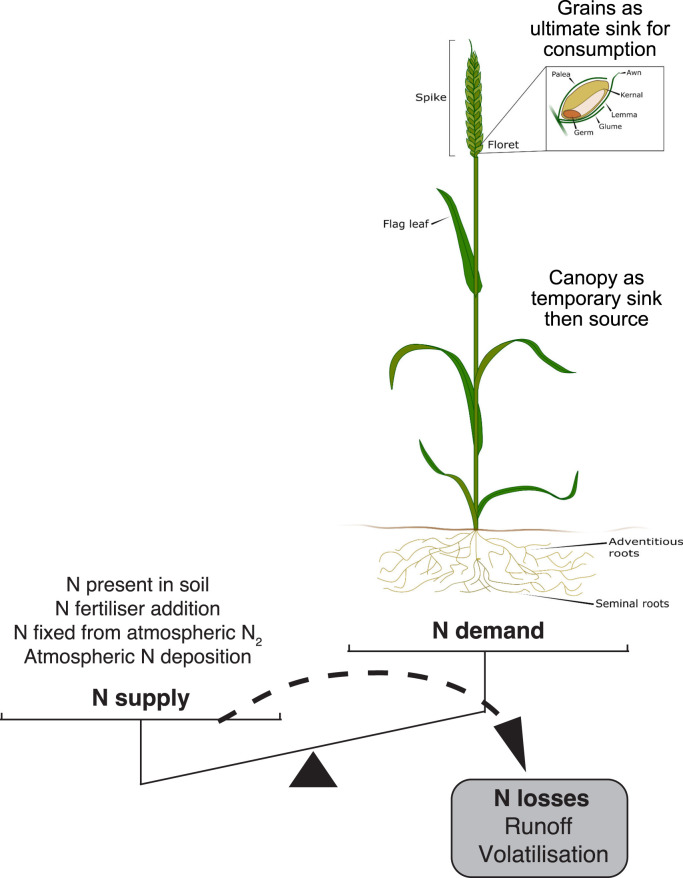
Lowering wheat N requirement as a mean to balance N supply and demand. A lack of equilibrium between N supply and crop N demand (both in terms of canopy production and grains) lead to N losses in the form of volatilisation and runoff, both causing major environmental issues. N demand varies throughout wheat development and must be matched by N supply to limit losses. Grains number, size, and protein content are the main drivers of N requirement.

Wheat (*Triticum aestivum* L.) is a major crop grown worldwide, with >700 million tonnes produced per year (FAO stat 2018), and accounting for 23% of the protein in people's diet [1]. Grain protein content (GPC), of which gluten protein accounts for 70–75%, is one of the main criteria determining grain quality. Sufficient nutrient inputs, nitrogen (N) in particular, are required by the developing plants to achieve both high yield and GPC. Limited N availability is a major issue in many agro-ecosystems as wheat plants do not establish symbiotic association with N_2_ fixing microbes. This limitation has been addressed by the development and widespread adoption of synthetic N fertilisers arising from the Haber–Bosch process. However, these are costly to farmers in developed countries, and remain unaffordable unless subsidised in developing countries, although their application is widespread and continues to increase [2]. The extensive use of synthetic fertiliser in wheat production, and lack of equilibrium between N supply and demand (Figure 1), has contributed to many environmental issues such as eutrophication of aquatic ecosystems [3], volatilisation of N_2_O (a potent greenhouse gas, [4,5]) and increases in N deposition in natural terrestrial ecosystems contributing to the loss of biodiversity [6]. There is, therefore, a pressing need to identify means (e.g. agronomic practises or new varieties) to produce wheat that meets quality requirements for human consumption while lowering the overall application and loss of synthetic N fertiliser. The development of new wheat genetic material either through breeding or genetic modification can offer opportunities to lower wheat N requirement, which will reduce the need for N application. However, these approaches require an understanding of the genetic and physiological determinants of low N requirement in wheat. In this review, we dissect the drivers of N requirement at the plant level and the temporal dynamics of supply and demand. We also discuss the potential for achieving plasticity in wheat N response and present evidence supporting tractable reductions in N requirement. Further opportunities to optimise microbial interactions to boost N supply are also discussed. Together, this provides a novel perspective on the ability to use physiological and genetic mechanisms to reduce wheat N inputs while maintaining productive potential.

## What are the drivers of wheat N requirement?

As the harvested and consumed component, wheat grains represent the ultimate N sink relevant to food production. Wheat grains are arranged on inflorescences (also known as spikes) with one inflorescence per tiller. Inflorescences are composed of an unbranched central rachis surrounded by single spikelets on either side, with each spikelet typically holding 3–4 fertile florets, and each floret potentially generating a grain (Figure 1). The number of spikes per plant, grains per spike and individual grain weight (see [7] for genetic determinant of wheat grain weight), determine the overall grain yield and potential N sink. Protein bodies located throughout the starchy endosperm of the wheat grain accumulate throughout grain development, starting from about 5 days post-anthesis. Endosperm proteins, particularly high molecular mass glutenins, are important for breadmaking characteristics. The germ (or embryo) appears to hold a higher N percent than the endosperm but represents an overall smaller sink [8]. Together, yield (number and size of grain) and GPC set the minimum N requirement for wheat production. However, these are negatively correlated, and increasing yield levels in newly selected varieties have been found to be associated with lower GPC in the U.K. [9], Germany [10], and the U.S. [11]. Recommendations of greater N application to achieve both high yield and GPC in varieties with greater N sink and lower predicted GPC have driven increases in N fertiliser use until the end of the last century [12]. While recent breeding efforts have increased yield at the expense of GPC, heritable variation in the positive grain protein deviation (GPD) from this negative correlation exists [13] and is associated with post-anthesis N uptake [14,15]. Therefore, selection for GPD may enable high yields with decreased N requirements.

Structural tissues maintaining the grains (palea, lemma, glumes, rachis in the spike, Figure 1 inset), leaves, stems, and roots hold the remainder of the N and constitute temporary N sinks throughout wheat plant development. During the vegetative stages (e.g. seedling development, tillering) leaf tissues represent a large N sink, which switches when the developing inflorescence emerge from the flag leaf sheath and especially after flowering (or anthesis) during the grain filling period. At anthesis, on average 23% of N (from above-ground tissues) is found in the ear with the remaining 35% in leaf blades, 14% in leaf sheath, and 28% in stem [16]. At maturity, an average of 89% of N (from above-ground tissues) is found in the ear with the remaining 5% in leaf blades, 6% in leaf sheath, and 4% in stem [16]. Some amount of N is also locked in roots, which are more difficult to assess in soil-grown plants. For example, wheat roots hold ∼2% N in younger plants (tillering stage), which decreases as the plant matures [17]. While lowering the N requirements relating to grain yield and protein content is problematic and could jeopardise productivity, manipulating N requirement related to these transitional N sinks offer an attractive alternative for improvements. These alternatives could either relate to the amount of N used to achieve specific development of transitional roles, or to the efficiency of the remobilisation to the grains at maturity.

One of the improvements brought about by the Green Revolution was an increase in the ratio of grain biomass to above-ground biomass (i.e. harvest index, HI), a result of selection for semi-dwarf traits for which underlying genes have been identified ([18], and references therein). Shorter wheat varieties partition less assimilates (carbon, C, and N) to the stem, but more to the grain and are more resistant to lodging (the physical displacement of the stem that can impair efficient harvest). In more recent varieties HI has reached ∼50–55% for wheat (for review [19]), with little improvement in the last 20 years, while recent increases in yield have been achieved through improved source development during the pre-anthesis foundation phase [20]. New reduced height (*Rht*) alleles are being sought [21]. Besides providing structural support to the spike, the stem may also hold significant amount of assimilates especially during the grain filling phase [16]. Thus, simply decreasing the size of transitory sink tissues is not sufficient, and the processes that lead to uptake, assimilation, and remobilisation from source tissues to sink tissues must be efficient.

## Matching N uptake to N demand

N requirement varies with wheat developmental stage [22]. While wheat seedlings rely on the seed N store in the early developmental stages, high N levels are necessary for seedling growth, tiller production, and the establishment of an efficient canopy that can ensure sufficient C and N assimilation. Nitrogen taken up at the root level tends to be transported to the shoot for assimilation into amino acid through the glutamine synthetase-glutamine oxoglutarate aminotransferase (GS-GOGAT) pathway (for review [23]).

Wheat takes up N primarily in the form of nitrate (${\text{NO}}_{3}^{-}$) and ammonium (${\text{NH}}_{4}^{+}$) from the soil solution (amino acids can also be taken up but contribute less to the total N nutrition especially under high N conditions, [24]). These two N molecules can be provided as NH_4_NO_3_ fertiliser or be derived from soil organic matter (if urea is provided as N fertiliser it is reduced in the soil, a process that also leads to some amount of NH_3_ volatilisation). A suite of low and high-affinity transporters are necessary to take up N from the soil solutions and the genes encoding these have now been identified in wheat [25,26]. At the uptake level, nitrate and ammonium transporters need to show high affinity, being present in abundance (and continuing to show high affinity even under higher N conditions). At the root level, N can also be released in the form of root exudates (as amino acid, [27]), thus there is some level of N recycling at the root level.

Investigating the physiological underpinning of high GPD led Bogard et al. [14] to identify a positive correlation between post-anthesis N uptake and GPD. Post-anthesis N uptake can contribute from 5% to 40% of the total grain N in winter wheat and is dependent on N being available in the soil during the grain filling period as well as favourable climatic conditions [28]. So far, the physiological determinants of post-anthesis N uptake are not well understood and merit further study given the potential opportunity to improve GPC without affecting yield. Taulemesse et al. [15] showed that under high N conditions post-anthesis N uptake per g dry weight was reduced compared with low N availability conditions. The physiological functioning of post-anthesis N uptake remains uncovered as it is not clear whether the N taken up by the roots post-anthesis is directly transported to the developing grain, thereby bypassing the flag leaf. Some level of nitrate reductase activity (a necessary step in the process of assimilating nitrate into amino acid) has been detected in both the grain and surrounding tissues (glumes, [29]). Therefore, there is evidence to suggest value in further work to elucidate the role of different tissues (root, shoot, leaves) in post-anthesis N uptake and transport and in further understanding the associated molecular responses.

## Coordination of C and N metabolic processes

Leaf blades represent the largest N sink during vegetative development before becoming a N source during the grain filling period and leaf senescence. Within the leaves, the most abundant protein is rubisco (ribulose 1,5-bisphosphate carboxylase/oxygenase), which carboxylates ribulose 1,5-bisphosphate (RuBP) a key step in photosynthetic C fixation. Rubisco represents 51% of leaf total N in wheat [21,22], though the total N cost of the entire photosynthetic apparatus is higher as light-harvesting complexes and photosystems also represent a high proportion of the C3 leaf N budget [30]. Given that rubisco has a low catalytic capacity (though on par with other similar catalytic enzymes), high N level is necessary to ensure high rubisco activity and therefore high CO_2_ fixation. Leaf N content is correlated with leaf-level CO_2_ assimilation rate; however, the slope of the correlation declines as N availability increases [31]. There is some level of variation in enzymatic characteristics of the rubisco protein from different members of the Triticeae family, suggesting that for a unit of N stored in the leaves greater photosynthetic fixation could be achieved [32].

In addition to carboxylation, rubisco can also oxygenate RuBP, which leads to the production of 3-phosphoglycerate (3-PGA) and 2-phosphoglycolate (2-PG). Due to the inhibitory activities of 2-PG, this metabolite is quickly converted through the photorespiratory pathway to 3-PGA. This process also generates CO_2_ and ${\text{NH}}_{4}^{+}$ in the mitochondria through the activity of glycine decarboxylase. ${\text{NH}}_{4}^{+}$ is subsequently assimilated in the chloroplast by the plastidic isoform of glutamine synthetase (GS2). Photorespiration has generally been considered a wasteful process and it has been proposed that productivity increases could be achieved if it can be by-passed in crop [33,34]. However, Busch et al. [35] showed that N assimilation through the photorespiratory pathway can provide an increase in CO_2_ fixation. An efficient assimilation in the leaf is important to prevent loss of NH_3_ through volatilisation, which can account for 1–4% of the applied N in a wheat field [36]. NH_3_ volatilisation occurs when the mole fraction of NH_3_ in the atmosphere is lower than the mole fraction of gaseous NH_3_ above the water film in the mesophyll cell walls in the substomatal cavity (NH_3_ compensation point), and NH_3_ is absorbed if it is higher [37–39]. Lower N availability [36] as well as low GS activity leads to higher NH_3_ compensation point, and volatilisation [38]. Dynamic processes associated with photosynthesis drive C fixation and N demand during plant development. Optimising N requirement requires further understanding of the specific coordination of processes.

## Increased N requirement with increased N availability

Besides being a necessary macronutrient, N (especially in the form of nitrate ${\text{NO}}_{3}^{-}$) plays a role as a signalling molecule [40]. For example, external availability of N to a plant otherwise depleted in N leads to over-proliferation of roots while overall high N availability leads to a more compact and restrained root architecture. In *Arabidopsis thaliana*, AtNRT1;1 has been proposed as a transceptor (protein that plays the role of both receptor and transporter, [41]), though the wheat ortholog of AtNRT1;1 has yet to be characterised. This poses the question as to whether a similar mechanism of N perception is conserved in wheat and highlights the importance of conducting similar work to better understand the signal transduction pathway associated with N requirement in wheat.

In addition to its role in shaping root architecture, N can also influence shoot architecture. Increased N availability can induce tiller number and tiller height [42]. Higher availability early in the season will induce tiller production and the potential size of the N sink at maturity. This can be problematic as tillers that emerge later during development compete for light and nutrients [43,44], and surplus late tillers tend to die back or be less productive than early tillers. This may ultimately lead to a lower yield per unit of N (also defined as N use efficiency, NUE) when plants are grown under high vs low N conditions [10,45,46].

To achieve low N requirement, many aspects of wheat plant metabolism, including uptake, assimilation and remobilisation steps must be co-regulated. Regulatory elements that can affect all steps in a co-ordinated manner are of interest. For example, Grf4 was shown to be a N-responsive transcriptional regulator promoting both ${\text{NH}}_{4}^{+}$ uptake and growth in response to N supply (and counteracting the inhibitory effects of the DELLA protein SLR1 in rice [47]). More recently, N-dependent chromatin modification was shown to regulate tillering in rice [48]. Similarly, phytohormones (e.g. strigolactones, cytokinins, brassinosteroids) have the capacity to modulate the N response and affect different aspects of growth, including sink capacity [49–51].

## Can N requirement be lowered in wheat?

Studies at the biochemical and physiological level in wheat tend to be conducted on modern varieties, which have been selected for a narrow specification of high yield and sufficient GPC under current agronomic practises including high N availability [10,52]. Changes in the commonly used bread making process meant that lower GPC is required and breeding for greater proportions of high molecular mass glutenin protein rather than gliadin in the grain [53] enabled good bread baking performance at lower overall GPC levels. Modern varieties, therefore, occupy a narrow range of yield and GPC. Yet, there is wide genetic and phenotypic diversity amongst wheat germplasm when landraces and crop wild relatives are included as well as elite varieties [10,11]. Many of these are adapted to low input environments suggesting that interesting alleles that may provide an advantage for growth under low N availability may be available but not yet exploited or incorporated into modern varieties.

As part of the Green Revolution, short straw varieties were selected which provided some level of protection against lodging. These varieties show a reduced N response in terms of shoot architecture (e.g. height, tillering capacity), due to low sensitivity to gibberellins [47,48]. Historic varieties also showed an altered root system architecture compared with modern varieties [54] and it seems that in terms of root plasticity [55], high N response has been selected for [56].

The fact that NUE is much lower for wheat plants grown under high N conditions compared with low N conditions suggests that wheat can be more efficient with the available N and that there is an unattained potential for lowering N requirement whilst maintaining high yield and GPC. Screening collections of landraces such as those held in the Watkins collection [57] for yield and GPC under a range of N conditions may provide a source of functional variation leading to lower N requirement.

In addition to the improvement to the wheat genetic material being used careful consideration of agronomic practices may compound some of the benefits. Studies of single plants growing in pots are very useful to establish N budget on a per plant basis and at varied developmental stages, but wheat plants are cultivated in fields within high-density populations (in the U.K. 350 plants.m^−2^ is quite common at establishment). It is well known that plants respond to the presence of neighbours both above and below-ground [58,59]. In particular, increasing planting density can regulate the tiller number and lead to lower N requirements [42] and together with a change in leaf angle can increase NUE (in rice [60]).

## Wheat-driven optimisation of the microbial community to maximise N availability

In the soil, wheat roots both compete with microbes for N uptake and also depend on these microorganisms for N availability. Wheat plants can manipulate or structure rhizosphere microbial communities, through the production of a myriad of root exudates [61,62]. Different rhizosphere communities can impact wheat yield through the production of hormones [63], and N availability. While efforts in developing cereals that can establish symbiotic association with N_2_ fixing microbes are a long-term goal [64], wheat association with endophytic N_2_ fixing microbes has shown that accessing alternative sources of N is feasible [65]. Selecting wheat varieties that can optimise their carbon usage to manipulate their associated microbial communities to maximise N availability would provide an avenue to lower the apparent N requirement for wheat production.

## Conclusion

Improving the efficiency of N use for wheat production has been a long-term goal, and thus far small incremental steps linked to yield increases have been achieved. Considering wheat N requirement provides new avenues for targeted improvement. Wheat plants with low N requirement should be able to perceive low amounts of N, respond effectively, and continue with a high level of productivity per unit if N supply even as N concentration increases. Overall, we propose that physiological and genetic approaches outlined here hold the potential to optimise the N fertiliser use for wheat production. In addition, Medici et al. [66] showed that in wheat as in other species such as *A. thaliana* and rice (*Oryza sativa*), N level controls the phosphate starvation response. This hints at a wider role for N in regulating additional nutrient efficiencies. Lowering N requirement may ultimately provide additional benefits to lowering overall nutrient requirements.

## Perspectives

- Application of synthetic nitrogen fertiliser for wheat production has significant economic and ecological cost. Understanding the determinant for low nitrogen requirement can provide new avenues for lowering the dependency on synthetic nitrogen fertilisers.
- N requirements are disproportionally high under high vs. low nitrogen availability suggesting that there is a potential yet unattained for lowering these N requirements.
- Understanding what regulates how responsive wheat plants can be to both external nitrogen availability and internal nitrogen status may lead to lowering nitrogen requirement.

## Open Access

Open access for this article was enabled by the participation of University of Cambridge in an all-inclusive *Read & Publish* pilot with Portland Press and the Biochemical Society under a transformative agreement with JISC.

## Abbreviations

2-PG: 2-phosphoglycolate
3-PGA: 3-phosphoglycerate
GPC: grain protein content
GPD: grain protein deviation
RuBP: ribulose 1,5-bisphosphate

## References

[1] Shewry, P.R. and Hey, S.J. (2015) The contribution of wheat to human diet and health. Food Energy Secur. 4, 178–202 10.1002/fes3.64 27610232 PMC4998136

[2] FAO (2018) World fertilizer trends and outlook to 2020. 1–38

[3] Herbert, R.A. (1999) Nitrogen cycling in coastal marine ecosystems. FEMS Microbiol. Rev. 23, 563–590 10.1111/j.1574-6976.1999.tb00414.x 10525167

[4] Shcherbak, I., Millar, N. and Robertson, G.P. (2014) Global metaanalysis of the nonlinear response of soil nitrous oxide (N_2_O) emissions to fertilizer nitrogen. Proc. Natl Acad. SCI. U.S.A. 111, 9199–9204 10.1073/pnas.1322434111 24927583 PMC4078848

[5] Bouwman, A.F., Boumans, L.J.M. and Batjes, N.H. (2002) Emissions of N_2_O and NO from fertilized fields: summary of available measurement data. Global Biogeochem. Cycles 16, 1058 10.1029/2001GB001811

[6] Simkin, S.M., Allen, E.B., Bowman, W.D., Clark, C.M., Belnap, J., Brooks, M.L. et al. (2016) Conditional vulnerability of plant diversity to atmospheric nitrogen deposition across the United States. Proc. Natl Acad. SCI. U.S.A. 113, 4086–4091 10.1073/pnas.1515241113 27035943 PMC4839424

[7] Brinton, J. and Uauy, C. (2019) A reductionist approach to dissecting grain weight and yield in wheat. J. Integr. Plant Biol. 106, 20115–20122 10.1111/jipb.12741 PMC6492019 30421518

[8] Ching, T.M. and Rynd, L. (1978) Developmental differences in embryos of high and low protein wheat seeds during germination. Plant Physiol. 62, 866–870 10.1104/pp.62.6.866 16660627 PMC1092243

[9] Scott, M.F., Fradgley, N., Bentley, A.R., Brabbs, T., Corke, F., Gardner, K.A. et al. (2020) Limited haplotype diversity underlies polygenic trait architecture across 70 years of wheat breeding. BioRxiv 10.1101/2020.09.15.296533 PMC8101041 33957956

[10] Voss-Fels, K.P., Stahl, A., Wittkop, B., Lichthardt, C., Nagler, S., Rose, T. et al. (2019) Breeding improves wheat productivity under contrasting agrochemical input levels. Nat. Plants 5, 706–714 10.1038/s41477-019-0445-5 31209285

[11] Maeoka, R.E., Sadras, V.O., Ciampitti, I.A., Diaz, D.R., Fritz, A.K. and Lollato, R.P. (2020) Changes in the phenotype of winter wheat varieties released between 1920 and 2016 in response to in-furrow fertilizer: biomass allocation, yield, and grain protein concentration. Front. Plant Sci. 10, 1786 10.3389/fpls.2019.01786/full 32082347 PMC7002544

[12] Muhammed, S.E., Coleman, K., Wu, L., Bell, V.A., Davies, J.A.C., Quinton, J.N. et al. (2018) Impact of two centuries of intensive agriculture on soil carbon, nitrogen and phosphorus cycling in the UK. Sci. Total Environ. 634, 1486–1504 10.1016/j.scitotenv.2018.03.378 29710647 PMC5981008

[13] Mosleth, E.F., Lillehammer, M., Pellny, T.K., Wood, A.J., Riche, A.B., Hussain, A. et al. (2020) Genetic variation and heritability of grain protein deviation in European wheat genotypes. Field Crops Res. 255, 107896 10.1016/j.fcr.2020.107896 32943810 PMC7397848

[14] Bogard, M., Allard, V., Brancourt-Hulmel, M., Heumez, E., Machet, J.-M., Jeuffroy, M.-H. et al. (2010) Deviation from the grain protein concentration–grain yield negative relationship is highly correlated to post-anthesis N uptake in winter wheat. J. Exp. Bot. 61, 4303–4312 10.1093/jxb/erq238 20679251

[15] Taulemesse, F., Le Gouis, J., Gouache, D., Gibon, Y. and Allard, V. (2015) Post-flowering nitrate uptake in wheat is controlled by N status at flowering, with a putative major role of root nitrate transporter NRT2.1. PLoS One 10, e0120291–23 10.1371/journal.pone.0120291 25798624 PMC4370649

[16] Barraclough, P.B., Lopez-Bellido, R. and Hawkesford, M.J. (2014) Genotypic variation in the uptake, partitioning and remobilisation of nitrogen during grain-filling in wheat. Field Crops Res. 156, 242–248 10.1016/j.fcr.2013.10.004 26412936 PMC4459691

[17] Mehta, K.M., Puntamkar, S.S. and Kalamkar, V.G. (1963) Study on uptake of nutrients by wheat as influenced by nitrogen and phosphorus fertilization. Soil Sci. Plant Nutr. 9, 29–34 10.1080/00380768.1963.10431052

[18] Hedden, P. (2002) The genes of the Green Revolution. Trends Genet. 19, 5–9 10.1016/s0168-9525(02)00009-4 12493241

[19] Foulkes, M.J., Slafer, G.A., Davies, W.J., Berry, P.M., Sylvester-Bradley, R., Martre, P. et al. (2010) Raising yield potential of wheat. III. Optimizing partitioning to grain while maintaining lodging resistance. J. Exp. Bot. 62, 469–486 10.1093/jxb/erq300 20952627

[20] Shearman, V.J., Sylvester-Bradley, R. and Scott, R.K. (2005) Physiological processes associated with wheat yield progress in the UK. Crop Sci. 45, 175–185 10.2135/cropsci2005.0175a

[21] Thomas, S.G. (2017) Novel *Rht-1* dwarfing genes: tools for wheat breeding and dissecting the function of DELLA proteins. J. Exp. Bot. 68, 354–358 10.1093/jxb/erw509 28201630 PMC5444474

[22] Zörb, C., Ludewig, U. and Hawkesford, M.J. (2018) Perspective on wheat yield and quality with reduced nitrogen supply. Trends Plant Sci. 23, 1029–1037 10.1016/j.tplants.2018.08.012 30249481 PMC6202697

[23] Forde, B. and Lea, P. (2007) Glutamate in plants: metabolism, regulation, and signalling 58, 2339–2358 10.1093/jxb/erm121 17578865

[24] El-Naggar, A., de Neergaard, A., El-Araby, A. and gh-Jensen H, H. (2009) Simultaneous uptake of multiple amino acids by wheat. J. Plant Nutr. 32, 725–740 10.1080/01904160902787842

[25] Guo, T., Xuan, H., Yang, Y., Wang, L., Wei, L., Wang, Y. et al. (2014) Transcription analysis of genes encoding the wheat root transporter NRT1 and NRT2 families during nitrogen starvation. J. Plant Growth Regul. 33, 837–848 10.1007/s00344-014-9435-z

[26] Buchner, P. and Hawkesford, M.J. (2014) Complex phylogeny and gene expression patterns of members of the NITRATE TRANSPORTER 1/PEPTIDE TRANSPORTER family (NPF) in wheat. J. Exp. Bot. 65, 5697–5710 10.1093/jxb/eru231 24913625 PMC4176842

[27] Kawasaki, A., Okada, S., Zhang, C., Delhaize, E., Mathesius, U., Richardson, A.E. et al. (2018) A sterile hydroponic system for characterising root exudates from specific root types and whole-root systems of large crop plants. Plant Methods 14, 114 10.1186/s13007-018-0380-x 30598690 PMC6300921

[28] Hirel, B., Le Gouis, J., Ney, B. and Gallais, A. (2007) The challenge of improving nitrogen use efficiency in crop plants: towards a more central role for genetic variability and quantitative genetics within integrated approaches. J. Exp. Bot. 58, 2369–2387 10.1093/jxb/erm097 17556767

[29] Abrol YP, S.K.M. and NTV, R. (1976) Nitrogen assimilation, its mobilization and accumulation in wheat (*Triticum aestivum* L.) grains. Cereal Res. Commun. 4, 431–440 https://www.jstor.org/stable/23778585

[30] Evans, J.R. and Clarke, V.C. (2018) The nitrogen cost of photosynthesis. J. Exp. Bot. 70, 7–15 10.1093/jxb/ery366 30357381

[31] Evans, J.R. (1983) Nitrogen and photosynthesis in the flag leaf of wheat (*Triticum aestivum* L). Plant Physiol. 72, 297–302 10.1104/pp.72.2.297 16662996 PMC1066227

[32] Prins, A., Orr, D.J., Andralojc, P.J., Reynolds, M.P., Carmo-Silva, E. and Parry, M.A.J. (2016) Rubisco catalytic properties of wild and domesticated relatives provide scope for improving wheat photosynthesis. J. Exp. Bot. 67, 1827–1838 10.1093/jxb/erv574 26798025 PMC4783365

[33] Maurino, V.G. (2019) Using energy-efficient synthetic biochemical pathways to bypass photorespiration. Biochem. Soc. Trans. 47, 1805–1813 10.1042/BST20190322 31754693

[34] South, P.F., Cavanagh, A.P., Liu, H.W. and Ort, D.R. (2019) Synthetic glycolate metabolism pathways stimulate crop growth and productivity in the field. Science 363, eaat9077–11 10.1126/science.aat9077 30606819 PMC7745124

[35] Busch, F.A., Sage, R.F. and Farquhar, G.D. (2018) Plants increase CO_2_ uptake by assimilating nitrogen via the photorespiratory pathway. Nat. Plants 4, 46–54 10.1038/s41477-017-0065-x 29229957

[36] Schjoerring, J. and Mattsson, M. (2001) Quantification of ammonia exchange between agricultural cropland and the atmosphere: measurements over two complete growth cycles of oilseed rape, wheat, barley and pea. Plant Soil. 228, 105–115 10.1023/A:1004851001342

[37] Husted, S. and Schjoerring, J.K. (1995) Apoplastic pH and ammonium concentration in leaves of *brassica napus* L. Plant Physiol. 109, 1453–1460 10.1104/pp.109.4.1453 12228682 PMC157681

[38] Husted, S. and Schjoerring, J.K. (1996) Ammonia flux between oilseed rape plants and the atmospher in response to changes in leaf temperature, light intensity, and air humidity. Plant Physiol. 112, 67–74 10.1104/pp.112.1.67 12226374 PMC157924

[39] Farquhar, G., Caemmerer, S. and Berry, J. (1980) A biochemical model of photosynthetic CO_2_ assimilation in leaves of C_3_ species. Planta 149, 78–90 10.1007/BF00386231 24306196

[40] Zhang, H. and Forde, B.G. (1998) An Arabidopsis MADS box gene that controls nutrient-induced changes in root architecture. Science 279, 407–409 10.1126/science.279.5349.407 9430595

[41] Gojon, A., Krouk, G., Perrine-Walker, F. and Laugier, E. (2011) Nitrate transceptor(s) in plants. J. Exp. Bot. 62, 2299–2308 10.1093/jxb/erq419 21239382

[42] Yang, D., Cai, T., Luo, Y. and Wang, Z. (2019) Optimizing plant density and nitrogen application to manipulate tiller growth and increase grain yield and nitrogen-use efficiency in winter wheat. Peer J. 7, e6484–e6426 10.7717/peerj.6484 30828492 PMC6396748

[43] Richards, R.A., Rebetzke, G.J., Condon, A.G. and van Herwaarden, A.F. (2002) Breeding opportunities for increasing the efficiency of water use and crop yield in temperate cereals. Crop Sci. 42, 111–112 10.2135/cropsci2002.0111 11756261

[44] Berry, P.M., Spink, J.H., Foulkes, M.J. and Wade, A. (2003) Quantifying the contributions and losses of dry matter from non-surviving shoots in four cultivars of winter wheat. Field Crops Res. 80, 111–121 10.1016/S0378-4290(02)00174-0

[45] Hawkesford, M.J. and Riche, A.B. (2020) Impacts of G x E x M on nitrogen use efficiency in wheat and future prospects. Front. Plant Sci. 11, 1157 10.3389/fpls.2020.01157/full 32903740 PMC7438886

[46] Hawkesford, M.J. (2014) Reducing the reliance on nitrogen fertilizer for wheat production. J. Cereal Sci. 59, 276–283 10.1016/j.jcs.2013.12.001 24882935 PMC4026125

[47] Li, S., Tian, Y., Wu, K., Ye, Y., Yu, J., Zhang, J. et al. (2018) Modulating plant growth–metabolism coordination for sustainable agriculture. Nature 560, 595–600 10.1038/s41586-018-0415-5 30111841 PMC6155485

[48] Wu, K., Wang, S., Song, W., Zhang, J., Wang, Y., Liu, Q. et al. (2020) Enhanced sustainable green revolution yield via nitrogen-responsive chromatin modulation in rice. Science 367, eaaz2046–11 10.1126/science.aaz2046 32029600

[49] Sun, H., Tao, J., Liu, S., Huang, S., Chen, S., Xie, X. et al. (2014) Strigolactones are involved in phosphate- and nitrate-deficiency-induced root development and auxin transport in rice. J. Exp. Bot. 65, 6735–6746 10.1093/jxb/eru029 24596173 PMC4246174

[50] Jia, Z., Giehl, R.F.H., Meyer, R.C., Altmann, T. and Wirén, N. (2019) Natural variation of BSK3 tunes brassinosteroid signaling to regulate root foraging under low nitrogen. Nat. Commun. 10, 2378 10.1038/s41467-019-10331-9 31147541 PMC6542857

[51] Jia, Z., Giehl, R.F.H. and Wirén von, N. (2020) The root foraging response under low nitrogen depends on DWARF1-mediated brassinosteroid biosynthesis. Plant Physiol. 183, 998–1010 10.1104/pp.20.00440 32398320 PMC7333712

[52] Fradgley, N., Gardner, K.A., Cockram, J., Elderfield, J., Hickey, J.M., Howell, P. et al. (2019) A large-scale pedigree resource of wheat reveals evidence for adaptation and selection by breeders. PLoS Biol. 17, e3000071–20 10.1371/journal.pbio.3000071 30818353 PMC6413959

[53] Shewry, P.R., Hassall, K.L., Grausgruber, H., Andersson, A.A.M., Lampi, A.M., Piironen, V. et al. (2020) Do modern types of wheat have lower quality for human health? Nutr. Bull. 8, 1813–1812 10.1111/nbu.12461 PMC7756780 33380903

[54] Fradgley, N., Evans, G., Biernaskie, J.M., Cockram, J., Marr, E.C., Oliver, A.G. et al. (2020) Effects of breeding history and crop management on the root architecture of wheat. Plant Soil 452, 587–600 10.1007/s11104-020-04585-2 32713967 PMC7371663

[55] Wojciechowski, T., Gooding, M.J., Ramsay, L. and Gregory, P.J. (2009) The effects of dwarfing genes on seedling root growth of wheat. J. Exp. Bot. 60, 2565–2573 10.1093/jxb/erp107 19439763 PMC2692010

[56] Melino, V.J., Fiene, G., Enju, A., Cai, J., Buchner, P. and Heuer, S. (2015) Genetic diversity for root plasticity and nitrogen uptake in wheat seedlings. Funct. Plant Biol. 42, 942–916 10.1071/FP15041 32480735

[57] Hawkesford, M.J. and Griffiths, S. (2019) Exploiting genetic variation in nitrogen use efficiency for cereal crop improvement. Curr. Opin. Plant Biol. 49, 35–42 10.1016/j.pbi.2019.05.003 31176099 PMC6692496

[58] Hortal, S., Lozano, Y.M., Bastida, F., Armas, C., Moreno, J.L., Garcia, C. et al. (2017) Plant-plant competition outcomes are modulated by plant effects on the soil bacterial community. Sci. Rep. 7, 17756 10.1038/s41598-017-18103-5 29259319 PMC5736699

[59] van Gelderen, K., Kang, C., Paalman, R., Keuskamp, D., Hayes, S. and Pierik, R. (2018) Far-red light detection in the shoot regulates lateral root development through the HY5 transcription factor. Plant Cell. 30, 101–116 10.1105/tpc.17.00771 29321188 PMC5810572

[60] Sakamoto, T., Morinaka, Y., Ohnishi, T., Sunohara, H., Fujioka, S., Ueguchi-Tanaka, M. et al. (2005) Erect leaves caused by brassinosteroid deficiency increase biomass production and grain yield in rice. Nat. Biotechnol. 24, 105–109 10.1038/nbt1173 16369540

[61] Chen, S., Waghmode, T.R., Sun, R., Kuramae, E.E., Hu, C. and Liu, B. (2019) Root-associated microbiomes of wheat under the combined effect of plant development and nitrogen fertilization. Microbiome 7, 36 10.1186/s40168-019-0750-2 31640813 PMC6806522

[62] Rossmann, M., Pérez-Jaramillo, J.E., Kavamura, V.N., Chiaramonte, J.B., Dumack, K., Fiore-Donno, A.M. et al. (2020) Multitrophic interactions in the rhizosphere microbiome of wheat: from bacteria and fungi to protists. FEMS Microbiol. Ecol. 96, e1002352–15 10.1093/femsec/fiaa032 32124916

[63] Wang, J., Li, R., Zhang, H., Wei, G. and Li, Z. (2020) Beneficial bacteria activate nutrients and promote wheat growth under conditions of reduced fertilizer application. BMC Microbiol. 20, 38 10.1186/s12866-020-1708-z 32085752 PMC7035779

[64] Oldroyd GED and Leyser O. (2020) A plant's diet, surviving in a variable nutrient environment. Science 368: eaba0196–eaba0199. 10.1126/science.aba0196 32241923

[65] Iniguez, A., Dong, Y. and Triplett, E. (2004) Nitrogen fixation in wheat provided by Klebsiella pneumoniae 342. Mol. Plant Microbe Interact. 17, 1078–1085 10.1094/MPMI.2004.17.10.1078 15497400

[66] Medici, A., Szponarski, W., Dangeville, P., Safi, A., Dissanayake, I.M., Saenchai, C. et al. (2019) Identification of molecular integrators shows that nitrogen actively controls the phosphate starvation response in plants. Plant Cell 31, 1171–1184 10.1105/tpc.18.00656 30872321 PMC6533016

